# Evaluation of the Dimension XL clinical chemistry system

**DOI:** 10.1155/S1463924697000023

**Published:** 1997

**Authors:** Neel V. Neelkantan, John P. Mizzer, Jack Weinacht, David R. Thompson, Bulent Sener, Diane K. Stille, Wei G. Zhao, David Miller

**Affiliations:** Dade International Chemistry Systems P.O. Box 6101 Glasgow 707 Newark DE 19714-6101 USA

## Abstract

The analytical performance of the Dimension XL clinical chemical system was evaluated. The XL is the latest addition to the Dimension family of instruments; it is a random access analyser with a throughput up to 740 tests/hour. Regression analysis of method comparison studies with Dimension AR yielded slopes of 0.93 to 1.03 and correlation coefficients ≥0.96 for 28 assays. Excellent precision performance was also observed. New instrument features of the XL are discussed.


Journal of Automatic Chemistry, Vol. 19, No. 1 (January–February 1997) pp. 9–13

### Evaluation of the Dimension® XL clinical chemistry system

**Neel V. Neelkantan*, John P. Mizzer, Jack Weinacht, David R. Thompson, Bulent Sener, Diane K. Stille, Wei G. Zhao and David Miller**

*Dade International, Chemistry Systems, P.O. Box 6101, Glasgow 707, Newark, DE 19714-6101.*

*The analytical performance of the Dimension® XL clinical chemical system was evaluated. The XL is the latest addition to the Dimension family of instruments; it is a random access analyser with a throughput up to 740 tests/hour. Regression analysis of method comparison studies with Dimension AR yielded slopes of 0·93 to 1·03 and correlation coefficients ≥0·96 for 28 assays. Excellent precision performance was also observed. New instrument features of the XL are discussed.*

### Introduction

The Dimension® clinical chemistry system is a general chemistry analyser introduced in 1985. The first version, the Dimension 380, with a throughput of 380 tests, was intended for smaller hospital laboratories. The next generation, Dimension AR, was introduced with a throughput of 500 tests/hour. The latest, the Dimension® XL clinical chemistry system, was recently introduced with a throughput of 740 tests/hour. The XL builds on continued enhancements in the areas of throughput, automation and ease of use.

The analytical equivalency with earlier models is reported in this paper and details of new instrument features are given. The evaluation included precision studies and split sample correlation of tests.

### Materials and methods

### *Instrumentation*

General instrumentation and operation of the Dimension systems have been described elsewhere [1–5]. New features on XL include automated reagent loading and unloading; segmented sample carriers, which allow intermixed barcoded and non-barcoded tubes and sample cups, along with paediatric sample utilization for more efficient work flow; automatic urine dilutions; and Integrated Multisensor Technology (IMT) for electrolytes. Urine dilutions are accomplished through the use of an on-board (self-contained) dilution wheel, while serum dilutions are done automatically in a cuvette. The system operation is controlled by a multi-processor computer system with an internal hard drive. The software provides such features as one-button daily maintenance and an enhanced quality-control (QC) program which stores 90 days of quality control on board with choice of Shewhart chart and Westgard rules. The on-board diagnostics automatically track cycles run on motors and parts. Parts are serviced and replaced during preventive maintenance before they fail. Remote diagnostic capability speeds troubleshooting via a built-in modem. The uninterruptable power supply keeps the XL sampling and processing for 10 minutes before beginning an automatic power-down, in the event of loss of power. All sample results and data are saved. Similar to the Dimension AR, the XL has two reagent arms capable of delivering multiple reagents to reaction cuvettes and also providing mixing through ultrasonics. Reaction cuvettes are automatically made on-board and sealed for disposal. Cuvette temperature is controlled to 37·0 + / − 0·1 °C. A rotating photometric arm provides readings at any given reaction cuvette. The operating cycle time was reduced from 12 s on the AR to 7·2 s on the XL to attain the photometric throughput of 500 tests/hour.

### *Integrated Multisensor Technology (IMT)*

The IMT system for measurement of electrolytes on the Dimension AR and XL systems was introduced recently. These methods are processed with an independent module from that of the photometric methods which is capable of 240 tests/hour, for the overall throughput of 740 tests/hour. A unique feature of the IMT system is a disposable sensor cartridge which incorporates ion-selective sensors for Na, K, and Cl on a ceramic substrate. The cartridge also contains its own reference sensor and an air/liquid detector. The sensor substrate is based on hybrid thick film technology consisting of layers of conductor and dielectric material and printed polymer sensors. A dual channel elastomeric flow path sits directly over the sensor substrate and directs the sample over the individual sensors and a reference fluid over the reference sensor. The sensor cartridge is inserted into a cartridge interface on the analyser, establishing electrical and fluidic connections; insertion and removal of the cartridge is similar to that of an audio cassette tape. Electrolyte results are generated using standard potentiometric techniques. The TCO2 measurement system, based on the classical pH electrode, is adjacent to the cartridge interface and has been integrated with the IMT system. The cartridge can be used for up to 400 patient samples or 48 hours (whichever comes first). When expired, the cartridge is replaced with a new one. No electrode maintenance is necessary. Current work is also in progress to allow separate processing of glucose, BUN and creatinine (currently photometric methods) via enzyme sensor technology. This will allow further enhancement in throughput to 920 tests/hour.

* Correspondence to N. V. Neelkantan, Tel: (302) 451-3660; fax: (302) 451-4998.

0142-0453/97 $12·00 © 1997 Taylor & Francis Ltd

### *Software*

The Dimension XL software consists of 30 interacting tasks. These tasks provide an interface with the user, maintain reagent inventories, schedule instrument activities and control the various instrument resources. The tasks are designed around a layered hierarchy, allowing for real-time control, sophisticated scheduling and future expansion.

The user's primary interaction with the Dimension XL software is the RUN key. After the RUN key has been pressed, the system will scan for new samples, query the host for test requests (or automatically set up a default panel or pre-entered request), allocate reagent and begin processing samples. If there are system needs, then the user will be prompted to address those needs.

Other Dimension XL software features include automatic urine dilution, automatic re-run of tests, programmable off-hour hydration, an on-board quality control package, one-button daily maintenance, remote access capability, and an on-board reliability-oriented maintenance system, which tracks part and motor usage. The XL contains an internal modem for use in remote troubleshooting. The modem allows Dade personnel and Dimension XL customers to exchange information. Secure access to the XL system is provided by password protection. With the appropriate software, the remote system supports a variety of hardware platforms. Currently, Dade interfaces to Dimension XLs using Macintosh Power PCs which are connected through a Xyplex server to a bank of 16 28·8 K baud modems. The remote users are shielded from needing to understand the operation of the XL's UNIX operating system through the use of UNIX shell scripts, the AWK programming language, and general UNIX user/group/world file and directory protection schemes. File compression and decompression are provided for the transfer of large information files.

### *Reliability*

Both hardware and software were monitored, calculated, evaluated and improved in a closed-loop-corrective-action environment from prototype to commercialization of the product. Early reliability predictions allowed for an improved selection of more robust components on critical parts. The key metrics of availability, mean cycles (tests) between failures (MCBF), failure rate, reliability-growth-slope and Laplace-Trend tests were periodically calculated to determine the progress of the instrument reliability during the product development cycle. Both the hardware and software failure modes encountered were analysed and eliminated. Finally, all the redesign activities were verified via software validation/verification and hardware reliability testing of the instrument to assure the effectiveness of corrective actions.

The instruments were subjected to an additional six months of post-development internal reliability testing under routine operating conditions to ensure the effectiveness of the design. Decisions on preventative maintenance and instrument service schedules were also made during this period. These efforts have resulted in the introduction of a product, which the authors believe, is optimized for field reliability, availability and customer satisfaction.

### *Reagents*

Reagents, calibrators and verifiers used in the methods reported in this paper are available from Dade Chemistry Systems, Inc., Newark, DE 19702.

### *Specimens*

Serum, plasma, cerebrospinal fluid and urine samples for the method comparison study were obtained from hospital patients and normal subjects. The analyte concentrations spanned the assay range of tests studied. Multiqual control sera were obtained from Ciba Corning Diagnostics Corporation, Oberlin, OH.

### *Experimental procedures*

*Methods comparison*: The performance of the methods reported here were compared with that of Dimension AR. Both models of Dimension used the same reagent cartridge for each method. Both AR and XL instruments were calibrated prior to conducting the method comparison study. Patient serum/plasma specimens were analysed on both AR and XL instruments. The samples were chosen to represent reference interval and assay range. The sample population included hemolysed, icteric and lipaemic samples.

*Precision*: Within-run and total precision were evaluated according to NCCLS protocol EP5-T2 by duplicate analysis of two levels of control product on 20 non-consecutive analysis days.

*Heterogeneous immunoassay*: This format is used for the digoxin assay. The heterogeneous immunoassay format used for digoxin assay is a modification of Dade Chemistry System's ACMIA (Affinity-Column-Mediated Immunometric Assay) technology [6]. The sample containing digoxin was mixed and incubated with the antibody-ßgalactosidase conjugate. To this mixture was added oubain coated chromium dioxide. Oubain is an analogue of digoxin. Free antibody-enzyme conjugate is retained by the particles while the bound digoxin-antibody-enzyme conjugate complex remains in solution. Separation was achieved by applying a magnetic field to this mixture. Chromium dioxide particles were pulled to the side of the cuvette while the supernatant was withdrawn and added to a second cuvette containing the substrate chlorophenol red-ß-D-galactopyranoside (CPRG). The change in absorbance at 577 nM over a defined time interval, due to the formation of chlorophenol red, is directly proportional to digoxin concentration. The apparatus and method for reagent separation used in this assay are described in a patent application [7].

### Results and discussion

### *Assay performance*

Dimension XL assay performance of methods was compared with that of Dimension AR; the results are shown in table 1. The representative assays indicated in table 1

*Table 1. Split sample correlation (comparison method—Dimension AR).*

| *Analyte* | *Units* | *N* | *r* | *Slope* | *y-intercept* | $S_{yx}$ | *% TAE* |
|---|---|---|---|---|---|---|---|
| Total protein | g/l {g/dl} | 64 | 0·971 | 0·962 | 3·4 {0·34} | 1·9 {0·19} | 2·4 |
| Albumin | g/l {g/dl} | 50 | 0·997 | 1·0 | 1·3 {0·13} | 0·9 {0·09} | 2·57 |
| Total bilirubin | µmol/l {mg/dl} | 72 | 0·999 | 1·03 | 0·62 {0·036} | 4·1 {0·24} | 3·02 |
| Iron | µmol/l {µg/dl} | 34 | 0·996 | 1·04 | −0·21 {−1·19} | 1·1 {6·15} | 7·22 |
| Ammonia | µmol/l | 41 | 0·999 | 0·99 | 3·28 | 9·69 | 14·30 |
| Calcium | mmol/l {mg/dl} | 33 | 0·981 | 0·93 | 0·18 {0·70} | 0·05 {0·18} | 0·71 |
| CKMB | U/l | 87 | 0·996 | 0·99 | −1·4 | 3·25 | 8·67 |
| CK | U/l | 38 | 1·0 | 0·99 | 0·59 | 4·27 | 1·86 |
| Lipase | U/l | 40 | 0·998 | 1·0 | 2·36 | 21·6 | 5·89 |
| LDH | U/l | 48 | 1·0 | 0·98 | −1·94 | 2·48 | 4·46 |
| Alkaline phosphatase | U/l | 48 | 1·0 | 0·98 | −3·56 | 5·3 | 7·26 |
| Amylase | U/l | 39 | 0·999 | 0·983 | 0 | 1·07 | 0·64 |
| Cholesterol | mmol/l {mg/dl} | 49 | 0·998 | 1·01 | −0·02 {−0·69} | 0·12 {4·54} | 0·09 |
| Triglycerides | mmol/l {mg/dl} | 29 | 0·998 | 1·07 | −0.04 {−3·46} | 0·081 {7·14} | 3·37 |
| HDL | mmol/l {mg/dl} | 17 | 1·0 | 0·97 | 0·02 {0·59} | 0·02 {0·66} | 4·29 |
| Glucose | mmol/l {mg/dl} | 79 | 1·0 | 0·99 | 0·03 {0·59} | 0·2 {2·84} | 1·80 |
| Uric acid | µmol/l {mg/dl} | 29 | 0·995 | 1·0 | 7·7 {0·13} | 10·7 {0·18} | 0·26 |
| Creatinine | µmol/l {mg/dl} | 105 | 0·999 | 1·0 | 3·5 {0·04} | 10·6 {0·12} | 0·32 |
| BUN | mmol/l {mg/dl} | 106 | 0·994 | 1·0 | 0·03 {0·07} | 1·0 {2·73} | 3·69 |
| UCFP[a] | mg/l {mg/dl} | 49 | 0·999 | 1·0 | 0 | 15·4 {1·54} | 6·9 |
| Mg | mmol/l {mg/dl} | 112 | 0·992 | 0·94 | 0·09 {0·23} | 0·05 {0·13} | 1·52 |
| Phenobarbital | µmol/l {µg/ml} | 36 | 0·993 | 0·939 | 0·52 {0·12} | 5·95 {1·38} | 8·8 |
| DGNA[b] | nmol/l {ng/ml} | 24 | 0·999 | 0·989 | 0·06 {0·05} | 0·05 {0·04} | 10·19 |
| Sodium | mmol/l | 160 | 0·996 | 0·93 | 10·39 | 1·83 | 1·52 |
| Potassium | mmol/l | 163 | 0·998 | 1·01 | −0·02 | 0·05 | −0·39 |
| Chloride | mmol/l | 175 | 0·998 | 1·01 | 1·19 | 1·4 | 1·73 |
| TCO2 | mmol/l | 41 | 0·996 | 0·96 | 2·42 | 0·76 | 0·72 |

[a] = Urinary protein cerebrospinal fluid protein; [b] = Automated digoxin; TAE = total analytical error.

include general chemistries, enzyme assays, homogeneous immuno assays using Particle Enhanced Turbidimetric Inhibition immunoassay technology (PETINIA), Enzyme Multiplied Immunoassay technology (EMIT) and heterogeneous immunoassay. In all cases, the results on Dimension XL compared well with results on Dimension AR. The relative accuracy was assessed using the criteria of Westgard and Hunt [8].

Screening assays in urine for drugs of abuse methods are also part of the Dimension XL menu. The methods (amphetamine, barbiturates, cocaine metabolite, methadone, opiates, phencyclidines and cannabinoids) are adaptations of the Roche Abuscreen online methods. These utilize a particle based turbidimetric immunoassay technique in an all-liquid ready-to-use format. The qualitative methods are intended for the screening of urine samples with results expressed in units normalized to 1000 at the cut-off level. The absorbance change at the cut-off calibrator level for each method is normalized during calibration to provide a reference numerical value arbitrarily set to 1000. These units are referred to as 'normalized qual units'. Negative results are less than 1000 normalized qual units, and positive results are greater than or equal to 1000 normalized qual units. The instrument automatically calculates and prints the results in normalized qual units, as well as positive or negative.

### *Precision*

Table 2 summarizes the precision results. The precision performance is equivalent to that of Dimension AR (product literature).

The new features on XL add considerable value for the customer. The segmented sample wheel allows samples to be processed as they arrive in the laboratory, while the system is in operation, improving work flow and turnaround time. The sophisticated scheduling system is constantly looking at the samples in the system, optimizing both system throughput and ensuring quick STAT response. As soon as a STAT sample is introduced into the system, the scheduler will update and reorganize the pending tests to ensure prompt initiation of the STAT tests.

The programmable off-hour hydration automatically prepares reagents during off or slow shift periods. The reagent set-up screen allows the user to configure the number of tests that need to be hydrated for each method. In addition, the user can program a timer, up to a week in advance, that once activated will begin preparing reagents. This timer can be programmed to automatically activate every week, on selected or all days.

Remote diagnostics is a new feature on XL. Once connected to the system, a user can view all system screens and perform all system operations through a remote monitor and keyboard. This allows the user to diagnose instrument problems as well as chemistry problems. In addition to the screen viewing and keyboard entry capabilities, the user has the ability to either locally view or download additional troubleshooting and system information files. All patient specific data is automatically removed from files to provide for patient confidentiality.

*Table 2. Within-run precision (N = 20).*

| *Analyte* | *Units* | *Mean* | *Within-run SD (%CV)* | *Total SD (%CV)* |
|---|---|---|---|---|
| Total protein | g/l {g/dl} | Low 48·3 {4·83}<br>High 72·9 {7·29} | 0·4 {0·04}(0·8)<br>0·6 {0·06}(0·8) | 0·70 {0·07}(1·4)<br>0·70 {0·07}(1·0) |
| Total bilirubin | μmol/l {mg/dl} | Low 19·2 {1·12}<br>High 123·6 {7·23} | 0·17 {0·01}(0·88)<br>1·03 {0·06}(0·83) | 1·20 {0·07}(6·25)<br>1·88 {0·11}(1·52) |
| Uric acid | μmol/l {mg/dl} | Low 280·2 {4·71}<br>High 532·9 {9·0} | 9·52 {0·16}(3·4)<br>17·84 {0·30}(3·3) | 9·52 {0·16}(3·4)<br>17·84 {0·30}(1·52) |
| CK | U/l | Low 78·98<br>High 287·63 | 2·03 (2·6)<br>7·35 (2·6) | 3·80 (4·8)<br>14·37 (5·0) |
| Iron | μmol/l {μg/dl} | Low 13·1 {73·2}<br>High 34·2 {190·7} | 0·18 {1·0}(1·4)<br>0·25 {1·38}(0·7) | 0·31 {1·73}(2·4)<br>0·36 {2·04}(3·3) |
| LDH | U/l | Low 108·9<br>High 256·6 | 1·95 (1·8)<br>3·40 (1·3) | 3·50 (3·2)<br>4·96 (1·9) |
| Glucose | mmol/l {mg/dl} | Low 4·8 {86·9}<br>High 16·8 {302·8} | 0·06 {0·99}(1·2)<br>0·22 {3·93}(1·3) | 0·08 {1·41}(1·6)<br>0·28 {5·07}(1·7) |
| Cholesterol | mmol/l {mg/dl} | Low 3·3 {126·1}<br>High 7·3 {283·8} | 0·03 {1·27}(1·0)<br>0·12 {4·81}(1·6) | 0·05 {2·08}(1·7)<br>0·15 {5·78}(2·0) |
| Amylase | U/l | Low 73·79<br>High 464·44 | 0·85 (1·1)<br>6·48 (1·4) | 1·28 (1·7)<br>8·41 (1·8) |
| Phenobarbital | μmol/l {μg/ml} | Low 38·3 {8·90}<br>High 166·2 {38·57} | 2·4 {0·56}(6·3)<br>11·1 {2.57}(6·7) | 4·0 {0·92}(10·4)<br>18·75 {4·35}(11·3) |
| Automated digoxin | nmol/l {ng/ml} | Low 0·74 {0·58}<br>High 3·95 {3·08} | 0·03 {0·02}(3·5)<br>0·08 {0·06}(1·9) | 0·04 {0·03}(5·2)<br>0·18 {0·14}(4·5) |
| ALT | U/l | Low 40·19<br>High 181·33 | 1·47 (3·7)<br>2·13 (1·2) | 1·64 (4·1)<br>2·57 (1·4) |
| T4 | nmol/l {μg/dl} | Low 86·2 {6·70}<br>High 228·2 {17·7} | 4·0 {0·31}(4·6)<br>8·9 {0·69}(3·9) | 6·82 {0·53}(7·9)<br>9·91 {0·77}(4·3) |
| Tuptake | % | Low 37·39<br>High 42·95 | 0·74 (2·0)<br>0·54 (1·3) | 1·05 (2·8)<br>1·00 (2·3) |
| Theophylline | μmol/l {μg/ml} | Low 30 {5·4}<br>High 112·1 {30·2} | 0·61 {0·11}(2·1)<br>5·83 {1·05}(3·5) | 1·5 {0·27}(5·1)<br>6·83 {1·23}(4·1) |
| U. Amphetamine | qual. units | 500<br>1500 | 4·7 (0·5)<br>3·8 (0·36) | 8·2 (0·88)<br>9·8 (0·95) |
| U. Barbiturates | qual.units | 100<br>300 | 6·5 (0·74)<br>4·0 (0·37) | 10·2 (1·18)<br>7·4 (0·69) |
| U. Cannabinoids | qual/units | 25<br>75 | 3·84 (0·46)<br>3·23 (0·31) | 9·49 (1·14)<br>3·46 (0·33) |
| U. Phencyclidiines | qual.units | 12·5<br>37·5 | 6·9 (0·79)<br>18 (1·61) | 6·2 (0·69)<br>15·7 (1·42) |
| U. Opiates | qual.units | 150<br>450 | 7·59 (0·92)<br>21·1 (2·02) | 11·3 (1·4)<br>17·9 (1·73) |
| U. Methadone | qual.units | 150<br>450 | 10·31 (1·22)<br>12·41 (1·09) | 10·1 (1·18)<br>9·51 (0·83) |
| U. Cocaine | qual.units | 150<br>450 | 9·4 (1·04)<br>10·6 (1·00) | 7·5 (0·82)<br>12·2 (1·15) |
| Chloride | mmol/l | Low 94·57<br>High 119·30 | 0·45 (0·5)<br>0·51 (0·4) | 1·59 (1·7)<br>1·92 (1·6) |
| Sodium | mmol/l | Low 124·38<br>High 154·80 | 1·41 (1·1)<br>1·47 (1·0) | 3·11 (2·5)<br>4·17 (2·7) |
| Potassium | mmol/l | Low 3·20<br>High 5·68 | 0·03 (1·0)<br>0·07 (1·2) | 0·08 (2·4)<br>0·17 (3·1) |

U = Urine samples.

### Conclusion

The XL demonstrates analytical equivalence to that of previous Dimension systems. It is a user-friendly system with many features for easy operation for a large hospital laboratory. The instrument is capable of assaying 52 analytes on a random access basis, with a throughput of 740 tests/hour. The system also has 10 open channel applications. The majority of the chemistries have a calibration stability of approximately 90 days, and the immunoassays have a calibration stability of at least 30 days. The XL design also serves as a suitable platform for further workstation consolidation, such as addition of heterogeneous assay formats and robotic sampling.

### Acknowledgements

Excellent assistance from J. M. Shrewsbury, Glenn E. Marvel, and Jerry R. Roope is acknowledged.

### References

1. Knight, D., Singer, R., White, J. M. and Fraser, C. G., *Clinical Chemistry*, **34** (1989), 1899.
2. Lifshitz, M. and DeCrese, R., *The Instrument Report* (1988).
3. Lifshitz, M. and DeCrese, R., *The Instrument Report,* **3** (1991), 4.
4. Lifshitz, M. and DeCrese, R., *The Instrument Report,* **4**, (1993), 5.
5. Alpert, N. L., *Clinical Instrument Systems*, **14** (1996), 5.
6. Freytag, J. W., Dickinson, J. C. and Tseng, S. Y., *Clinical Chemistry*, **30** (1984), 417.
7. Wang, Chi-Chin, McKeever, Robert T. and Salyers, Marshall, L., U.S. Patent No. 5,128,103 (July 1992). *Apparatus for Automatically Processing magnetic Solid Phase Reagents.*
8. Westgard, J. O. and Hunt, M. R., *Clinical Chemistry*, **19** (1973), 49.

